# Supplementary material for: Integrative vectors for regulated expression of SARS-CoV-2 proteins implicated in RNA metabolism
Source: Wellcome Open Res. 2020 Nov 3;5:261. [Version 1] doi: 10.12688/wellcomeopenres.16322.1 (PMC7721065; doi:10.12688/wellcomeopenres.16322.1)
Supplement: Peer review history

# Peer review: Reviewer response for version 1

**DOI:** 10.21956/wellcomeopenres.17943.r41430

**Published:** 2020-12-03

**Authors:** Chad Petit

**Affiliations:** 1 Department of Biochemistry and Molecular Genetics, University of Alabama at Birmingham, Birmingham, AL, USA

The manuscript is well written with the development of the methodology clearly stated and presented to allow maximum impact for the scientific community. The etiological agent of the COVID-19 pandemic is the Severe Acute Respiratory Syndrome (SARS) coronavirus (CoV) 2. Unfortunately, the specific pathways and host-pathogen interactions that facilitate the viral lifecycle for this novel virus are largely not understood. The authors of this manuscript have selected 14 SARS-CoV-2 proteins that are expected to interact with RNA or RNA binding proteins for construction of reagents that can be used for analyzing their role in the coronavirus lifecycle. Briefly, numerous cell lines were established that express various forms (e.g. amino tagged, carboxyl tagged, etc) of each of the viral proteins. Furthermore, the expression of the viral proteins can be tuned to their relative expression levels during viral replication using different concentrations of doxycycline or incubation time.

 The rationale for the development of these reagents is sound in that they could be used for future research to understand how these viral proteins affect the host cell. The description of the synthesis of the reagents is clear and provided at a level of details that would allow the replication of the method by other labs. The conclusions about the method and their relevance for future deployment in other research programs are more than adequately supported by the data presented in the manuscript.

Is the rationale for developing the new method (or application) clearly explained?

Yes

Is the description of the method technically sound?

Yes

Are the conclusions about the method and its performance adequately supported by the findings presented in the article?

Yes

If any results are presented, are all the source data underlying the results available to ensure full reproducibility?

Yes

Are sufficient details provided to allow replication of the method development and its use by others?

Yes

Reviewer Expertise:

Structural virologist focusing on influenza and SARS-CoV-2.

I confirm that I have read this submission and believe that I have an appropriate level of expertise to confirm that it is of an acceptable scientific standard.

# Peer review: Reviewer response for version 1

**DOI:** 10.21956/wellcomeopenres.17943.r41258

**Published:** 2020-11-19

**Authors:** Sheila Graham

**Affiliations:** 1 MRC - University of Glasgow Centre for Virus Research, Institute of Infection, Immunity and Inflammation, College of Medical Veterinary and Life Sciences, University of Glasgow, Glasgow, UK

This is a methods paper that creates, and experimentally assesses, 54 inducible expression constructs encoding SARS-CoV2 (SCV2) proteins which are predicted to bind RNA. Bresson *et al.* carefully describe generation of this important resource, which will facilitate studies into how the host RNA proteome is affected by SCV2 infection and how viral proteins might alter RNA-protein interactions. Such a resource is essential for any thorough analysis of the SCV2 life cycle.

 In this method, an important innovation was a fix to get around use of RT-PCR, which uses an error-prone polymerase, to produce mutation-free constructs. Expression plasmids were designed to be multifunctional. They are suitable for transient expression, or hygromycin-selectable stable expression, following flippase integration into the genome of cells engineered to contain a flippase recombination target sequence. Finally, the constructs express proteins that are tandem affinity-tagged (untagged, or N- or C-terminally tagged) in order to facilitate purification. Importantly, the constructs were also codon optimised to ensure optimal expression levels.

 The well-thought out cloning strategy is well illustrated in Figure 2 allowing the method to be easily replicated. Quantification of expression levels as a percentage of total cellular protein is shown using a mass spectroscopy approach while western blots show the quality of expression and a time course of tetracycline induction. This is important to allow the reader to assess usefulness of the constructs.

 The article is scientifically sound. However, it would be helpful if the authors could suggest the nature of the cross reacting protein that they have used as a loading control in many of the western blots. They do not state the antibody used for the western blots in the figure legend. I imagine it is an anti-FLAG antibody?

 It would be good to be clear about the FH/HF nomenclature in the text and in Table 2 and the figure legends. Also correcting the misspelled “cloning” in the Figure would be good.

 It is a pity that Nsp12, encoding the catalytic subunit of the viral RdRp, a key RNA binding viral protein, was relatively poorly expressed, especially the C-terminal-tagged version. Can the authors give any information on their attempts to improve expression of this or any other viral proteins?

Is the rationale for developing the new method (or application) clearly explained?

Yes

Is the description of the method technically sound?

Yes

Are the conclusions about the method and its performance adequately supported by the findings presented in the article?

Yes

If any results are presented, are all the source data underlying the results available to ensure full reproducibility?

Yes

Are sufficient details provided to allow replication of the method development and its use by others?

Yes

Reviewer Expertise:

Virology, RNA biology.

I confirm that I have read this submission and believe that I have an appropriate level of expertise to confirm that it is of an acceptable scientific standard.
